# Low retinoic acid levels mediate regionalization of the Sertoli valve in the terminal segment of mouse seminiferous tubules

**DOI:** 10.1038/s41598-020-79987-4

**Published:** 2021-01-13

**Authors:** Kasane Imura-Kishi, Aya Uchida, Naoki Tsunekawa, Hitomi Suzuki, Hinako M. Takase, Yoshikazu Hirate, Masami Kanai-Azuma, Ryuji Hiramatsu, Masamichi Kurohmaru, Yoshiakira Kanai

**Affiliations:** 1 Department of Veterinary Anatomy, The University of Tokyo, Bunkyo-ku, Tokyo, Japan; 2 Department of Experimental Animal Model for Human Disease, Center for Experimental Animals, Tokyo Medical and Dental University, Bunkyo-ku, Tokyo, Japan

**Keywords:** Developmental biology, Stem cells

## Abstract

In mammalian testes, undifferentiated spermatogonia (A_undiff_) undergo differentiation in response to retinoic acid (RA), while their progenitor states are partially maintained by fibroblast growth factors (FGFs). Sertoli valve (SV) is a region located at the terminal end of seminiferous tubule (ST) adjacent to the rete testis (RT), where the high density of A_undiff_ is constitutively maintained with the absence of active spermatogenesis. However, the molecular and cellular characteristics of SV epithelia still remain unclear. In this study, we first identified the region-specific AKT phosphorylation in the SV Sertoli cells and demonstrated non-cell autonomous specialization of Sertoli cells in the SV region by performing a Sertoli cell ablation/replacement experiment. The expression of *Fgf9* was detected in the RT epithelia, while the exogenous administration of FGF9 caused ectopic AKT phosphorylation in the Sertoli cells of convoluted ST. Furthermore, we revealed the SV region-specific expression of *Cyp26a1*, which encodes an RA-degrading enzyme, and demonstrated that the increased RA levels in the SV region disrupt its pool of A_undiff_ by inducing their differentiation. Taken together, RT-derived FGFs and low levels of RA signaling contribute to the non-cell-autonomous regionalization of the SV epithelia and its local maintenance of A_undiff_ in the SV region.

## Introduction

In the convoluted seminiferous tubule (ST) of mammalian testes, undifferentiated (i.e., stem/progenitor) spermatogonia (A_undiff_) reside in the basal compartment, whereas differentiated spermatogonia (A_diff_) undergo meiotic differentiation and translocate into the adluminal compartment by moving across the tight junctions between adjacent Sertoli cells, followed by the progressive formation of spermatocytes, spermatids, and spermatozoa^1^. The spermatozoa are finally released into the lumen (i.e., spermiation), subsequently transferred by luminal fluid flow into the rete testis (RT), anastomosing tubules connecting to the efferent duct^2^.

The balance between maintenance and differentiation of germ line stem/progenitor cells is mainly coordinated by Sertoli cells through their expression of several signaling factors, such as members of the fibroblast growth factor (FGF), transforming growth factor-β (TGF-β) families, and retinoic acid (RA)^3–5^. In mouse testes, sex differentiation and maintenance of male germ cells are regulated by FGF9 and Nodal/Activin signals at the fetal and postnatal stages^6–10^. After sexual maturation, spermatogonial stem/progenitor cells are maintained mainly by glial cell line-derived neurotrophic factor (GDNF) signals and FGF2/FGF8/FGF5 within the basal compartment^11–14^. On the other hand, a subpopulation of A_undiff_ expresses RA receptors (RARs)^15,16^, and RA signals promote the differentiation of A_undiff_ into A_diff_^17,18^. In the convoluted ST, the levels of GDNF and RA are inversely correlated, and are cyclically regulated in a seminiferous epithelial cycle stage-dependent manner: high GDNF signal at early stages of the seminiferous epithelial cycle (reaching a peak around stage I)^19,20^ versus high RA signal at later stages (a peak at stage VIII)^21–23^. This balance between counteracting GDNF and RA signals appears to be involved in the continuous spermatogenesis in the convoluted ST of adult testes.

In the proximal terminal end of ST, the Sertoli valve (SV), a valve-like structure on the luminal side of the tubuli rectus, is formed at the border between the ST and RT in mammalian testis^24,25^. Such a structure in the terminal segment of ST may regulate the directional flow of the luminal fluid and sperm^24,25^, yet the exact function of the SV in spermatogenesis remains unclear. The SV epithelia lack most spermatogenic activity, mainly composed of GFRα1-positive stem/progenitor spermatogonia and proliferative Sertoli cells, which are biased to the proximal part of the SV region (SV niche)^26–28^. It is likely that certain unique signaling states within the SV epithelia (e.g., high GDNF expression) contribute to maintaining the GFRα1-positive A_undiff_ in the SV region^26,29^. However, the regionalization of the SV epithelia and their molecular/cellular characteristics are unclear.

In this study, we examined the postnatal development of the SV region and demonstrated the non-cell autonomous regionalization of the SV is due to active degradation of RA and, potentially, is modulated by FGF signaling from the adjacent RT region. We also investigated the molecular characteristics of SV epithelia by global mRNA analyses, comparing gene expression profiles of isolated SV fragments with those of ST and RT fragments.

## Results

### Constitutively activated AKT signals in the SV epithelia

**Figure 1:**
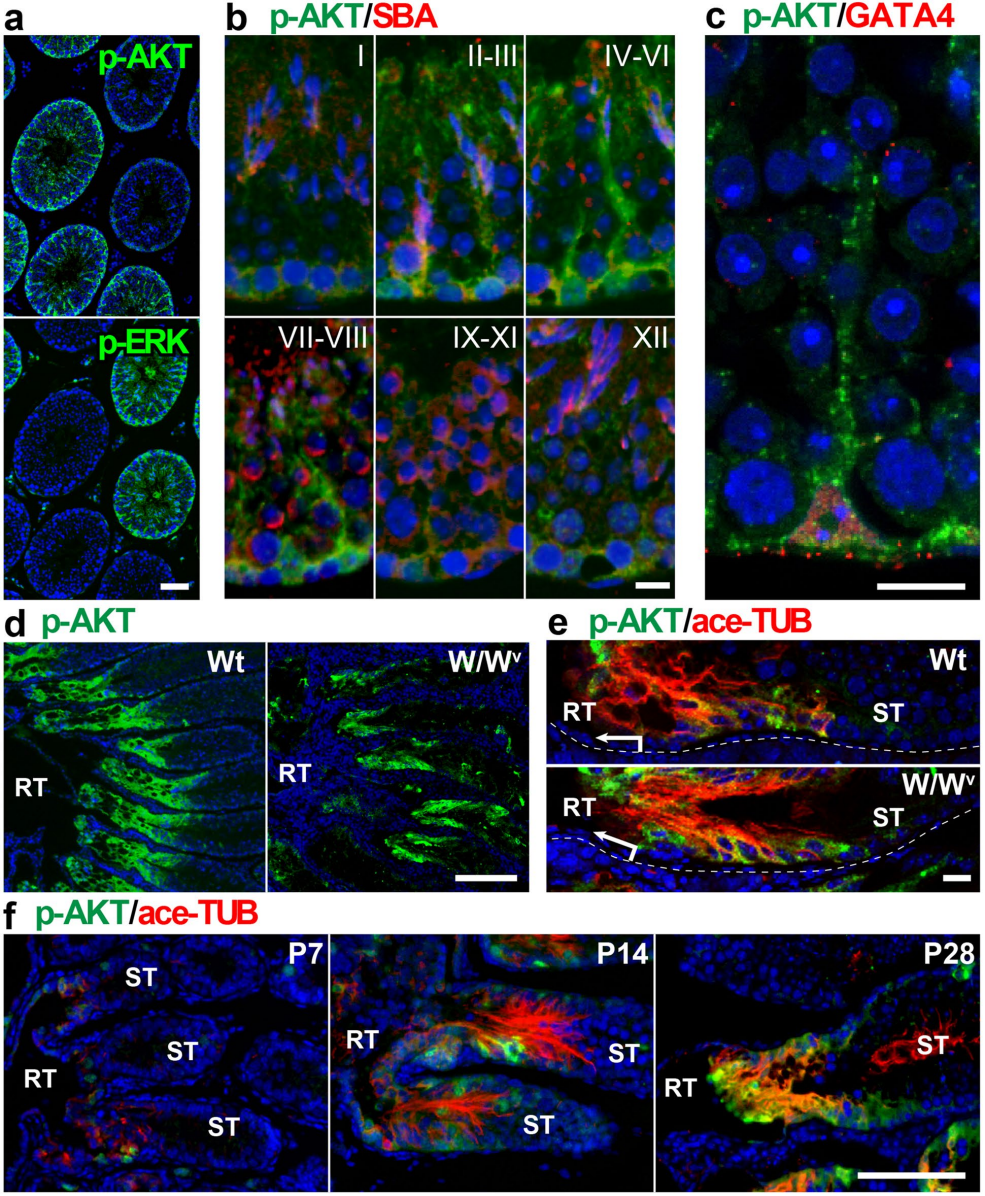
High phosphorylated AKT (p-AKT) signals in the SV epithelia. (**a**–**c**) Anti-p-AKT (green in **a**–**c**), anti-phosphorylated ERK (p-ERK; green in **a**), SBA lectin (acrosome staining; red in **b**), and anti-GATA4 (red in **c**) staining of the convoluted ST of adult wild-type mouse testes. In plate (**a**), anti-p-AKT and p-ERK immunostaining of two serial sections shows reversed complementary expression patterns of p-AKT and p-ERK in the ST. In plate (**c**), p-AKT signals were observed mainly in the cytoplasmic region of GATA4-positive Sertoli cells. In plate (**b**), roman numerals indicate the seminiferous epithelium cycle stage. (**d**,**e**) Anti-p-AKT (green) and anti-acetylated tubulin (ace-TUB; red) double staining of the proximal region of adult wild-type (Wt) and *W/W*^*v*^ testes, showing the transient region of RT and ST. Plate (**e**) shows the SV regions in plate (**d**) at higher magnifications. (**f**) Anti-p-AKT (green) and anti-ace-TUB (red) double staining of the proximal ST in the wild-type testes at postnatal (P) days 7, 14, and 28. RT, rete testis. ST, convoluted seminiferous tubules. Scale bars represent 50 μm in (**a**), 10 μm in (**b**,**c**), 200 μm in (**d**), 20 μm in (**e**), 100 μm in (**f**).

The SV epithelia in mouse testis comprise unique Sertoli cells with distinct characteristics from those in the convoluted seminiferous epithelia, represented by high-level expression of acetylated tubulin (ace-TUB)^26–29^. However, the mechanism of its regional specificity and homeostasis still remain to be discovered. In order to understand the specification of Sertoli cells within the SV region, we first screened the potential signaling pathways activated specifically in the SV epithelia and revealed constitutively high expression of phosphorylated AKT (p-AKT) in the Sertoli cells located within the SV region (Fig. 1a–f). In the convoluted seminiferous tubule (ST), p-AKT immunoreactivity was observed in the cytoplasm of Sertoli cells in a seminiferous epithelial cycle-dependent manner. p-AKT expression was high at seminiferous epithelial stages II–VI (Fig. 1b), whereas low at the seminiferous epithelial stages IX–XII (Fig. 1b), where the expression levels of phosphorylated ERK (p-ERK) are known to be high^30^. These data suggest the distinct regulation of AKT or ERK signaling pathway in the convoluted seminiferous epithelia with seminiferous epithelial stage dependency. Interestingly, Sertoli cells within the ace-TUB–positive SV region showed constitutively high expression levels of p-AKT expression, in comparison to the fluctuating expression pattern of p-AKT in the convolute seminiferous epithelia (Fig. 1d,e). In the developing postnatal mouse testes, the terminal segment of seminiferous tubules was partially positive for anti-p-AKT and ace-TUB staining at postnatal day (P) 7 (i.e., prior to the formation of the SV structure and the initiation of luminal fluid flow) (Fig. 1f). These signals became clearer in the Sertoli cells at P14, leading to the establishment of the SV structures with high expression of p-AKT by P28 (Fig. 1f). In the germ cell-depleted *W/W*^v^ mutant mice, p-AKT expression remained high in the SV region compared to the convoluted ST (“W/W^v^” in Fig. 1d,e), suggesting no appreciable contribution of spermatogenic cells to the high p-AKT expression in the SV epithelia (Fig. 1e). In contrast, p-ERK immunoreactivity was not restricted to the SV nor proximal ST regions, neither in wild-type and *W/W*^*v*^ testes (Fig. S1a). Signals of anti-phosphorylated p38 nor anti-phosphorylated JNK staining were not restricted to the SV region either (Fig. S1b). We further showed the ubiquitous expression of AKT1/2 in Sertoli cells of both ST and SV regions (Fig. S1c). The p-AKT signals were lost by the pretreatment with alkaline phosphatase prior to immunostaining, confirming the specificity of the anti-p-AKT antibody to the phosphorylated forms of AKT (Fig. S1d). Based on these findings, we conclude that p-AKT signals can be used as a marker of Sertoli cells in the SV region.

### Regeneration of the SV epithelia with AKT phosphorylation by the transplanted Sertoli cells

**Figure 2:**
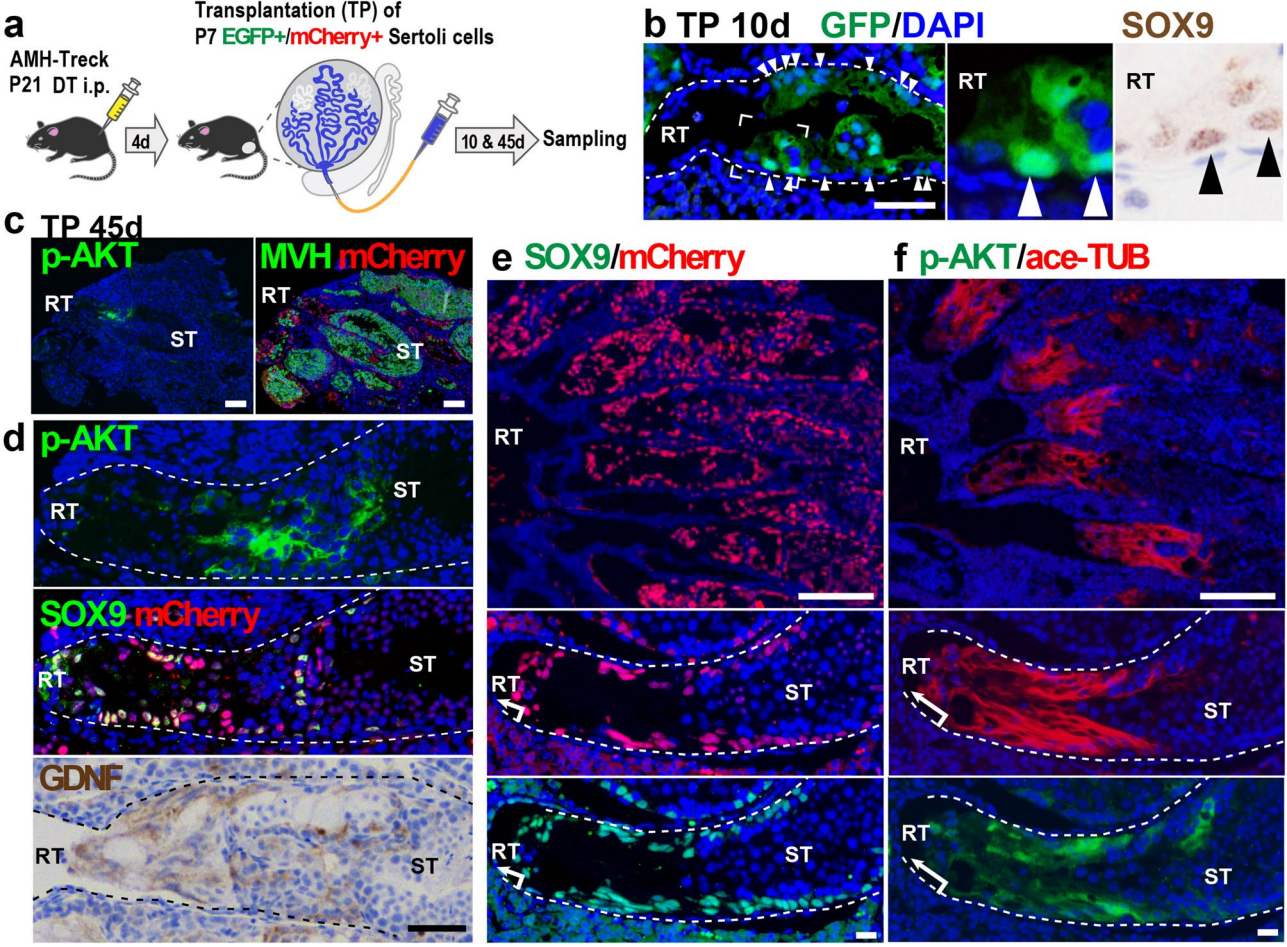
Regeneration of the SV epithelia by transplanted Sertoli cells adjacent to the RT. (**a**) Schematic representation of Sertoli cell ablation/transplantation experiment. GFP or mCherry-positive Sertoli cells (P7) were injected into the empty STs of recipient AMH-Treck males that were pretreated with DT at P21 (P21) to ablate endogenous Sertoli cells (n = 4). (**b**) Donor-derived (cytoplasmic/nuclear GFP-positive) Sertoli cells (arrowheads) were settled in the presumptive SV region adjacent to the host-derived (GFP-negative) RT in the recipient Tg male at day 10 post-transplantation. Insets, magnifications of two serial sections stained with anti-GFP (green) and anti-SOX9 (brown) antibodies respectively. (**c**–**f**) Anti-p-AKT, anti-SOX9, anti-MVH (germ cell marker), or anti-GDNF staining, together with anti-mCherry staining, in the proximal region of the Tg testes at day 45 post-transplantation. The donor-derived Sertoli (mCherry/SOX9-double positive) cells regenerated SV-like epithelia positive for anti-ace-TUB, anti-p-AKT, and anti-GDNF staining between the RT and the convoluted ST with several patches of active spermatogenesis (ST; MVH-positive in c; meiotic and post-meiotic germ cells in hematoxylin- and 4′,6-diamidino-2-phenylindole (DAPI)-stained images in **d**–**f**). Two panels of **c**, upper two panels of **d**, and two top and four lower panels of (**e**,**f**) represent serial sections. Broken lines are the outlines of the ST. *DT* diphtheria toxin, *RT* rete testis, *ST* seminiferous tubule. Scale bars represent 50 μm in (**b**,**d**), 100 μm in (**c**), 200 μm in (**e**,**f**) (upper), 20 μm in (**e**,**f**) (lower).

To examine whether the immature Sertoli cells adjacent to RT are pre-determined to form the SV epithelia or not, we conducted a Sertoli cell transplantation assay using the diphtheria toxin (DT)-treated AMH-Treck Tg male mice with Sertoli cell-ablated STs^31^. In brief, we collected Sertoli cell from the distal part of the convoluted STs (i.e., the region without RT and proximal ST) of CAG-EGFP (cytoplasmic/nuclear EGFP) or R26-H2B-mCherry (nuclear mCherry) mouse testes, and then injected the Sertoli cell suspension intratubularly into the DT-pretreated AMH-Treck host males as reported previously (Fig. 2a)^30^. At day 10 post-Sertoli cell transplantation, the donor-derived (GFP-positive) SOX9-positive Sertoli cells had settled in the presumptive SV regions adjacent to the host-derived RT in the recipient AMH-Treck Tg males (Fig. 2b), indicating the replacement of SV Sertoli cells with the transplanted donor-derived Sertoli cells. At day 45 post-transplantation, spermatogenesis was recovered in some of the proximal STs (Fig. 2c–f), in which the donor-derived (mCherry/SOX9-double positive) Sertoli cells had settled, including the presumptive SV region connecting to RT (Fig. 2d,e). Interestingly, the presumptive SV regions colonized by mCherry/SOX9-double positive transplanted Sertoli cells were also positive for anti-ace-TUB/p-AKT staining, reconstituting the valve-like structures enriched with GDNF expression (Fig. 2c,d,f). These findings imply that the SV structure was non-cell autonomously constructed by the transplanted Sertoli cells located adjacent to the RT, suggesting the possibility that the factors derived from the RT and its surrounding tissues contribute to the regionalization of mouse SV epithelia.

### Expression profile of FGF-related genes and potential contribution of FGF signaling to the AKT phosphorylation in Sertoli cells

**Figure 3:**
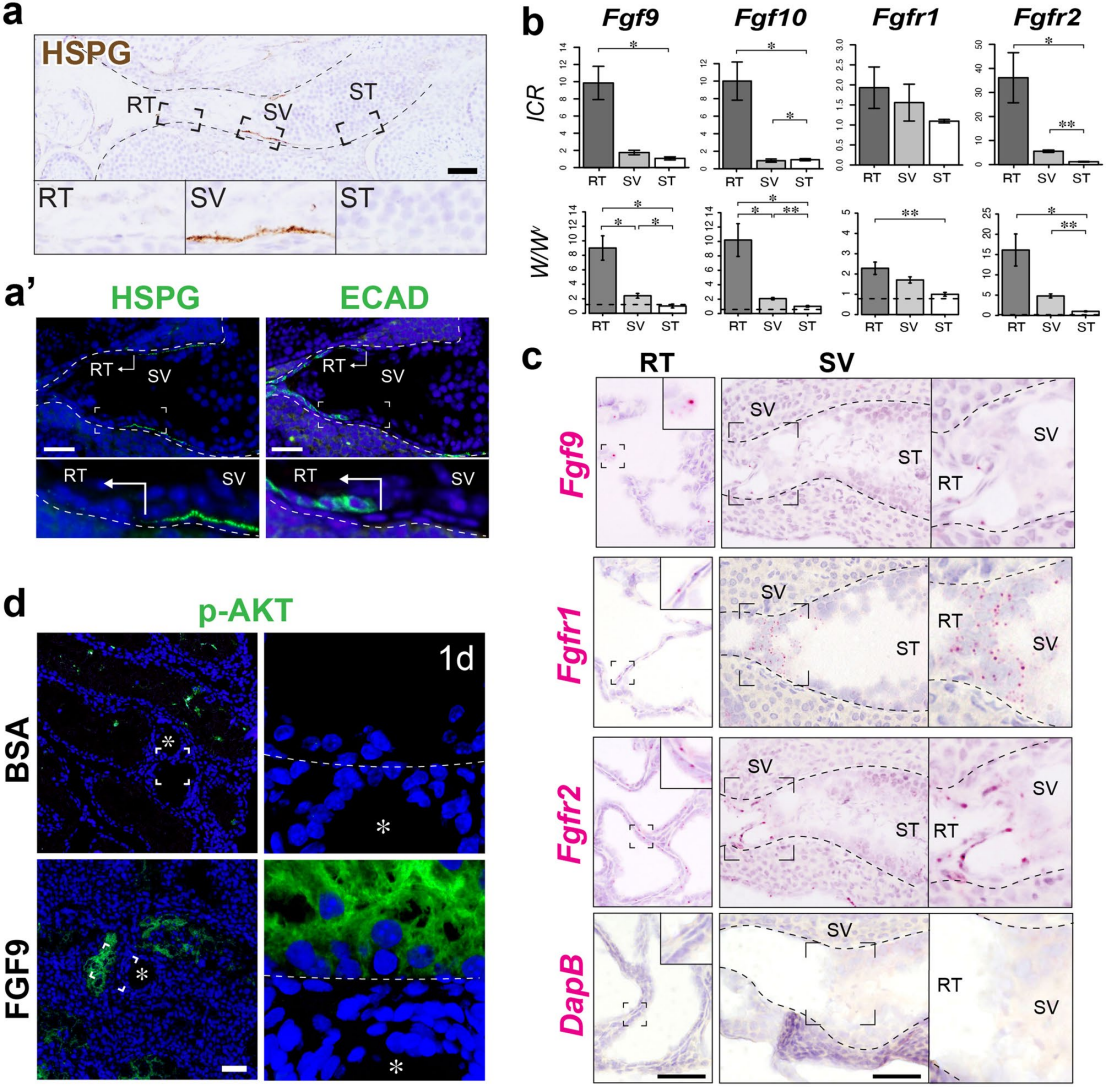
Expression profiles of FGF-associated genes and their association with p-AKT expression in the SV epithelia. (**a**) HSPG immunohistochemistry (brown in **a**, green in the left plate of **a’**), showing high HSPG expression in the basement membrane of the SV region, compared to those in the convoluted ST and the RT marked by ECAD (E-cadherin; Cadherin-1) immunohistochemistry (green in the right plate of **a’**). (**b**) Quantitative RT-PCR analysis of FGF-related genes (*Fgf9*, *Fgf10, Fgfr1* and *Fgfr2*) in the RT, SV, and convoluted ST fragments from wild-type ICR mouse testis (n = 4) and *W/W*^*v*^ testes (n = 5). Analysis was performed using a paired t-test. *p < 0.05; **p < 0.01. Broken horizontal line, expression level in the wild-type convoluted ST with active spermatogenesis. (**c**) In situ hybridization images of wild-type mouse testis, showing the expression of *Fgf9* in the RT, and expressions of *Fgfr1* and *Fgfr2* in both the RT and the SV. *DapB* was used as a negative control probe, showing little non-specific staining in the RT and the SV region. (**d**) Anti-p-AKT (green) staining of *W/W*^*v*^ mutant mouse testis at 24 h after FGF9-soaked bead transplantation, showing an ectopic appearance of p-AKT signals in the ST close to the transplanted FGF9-soaked bead (asterisk), not in the tubules far away from the bead or near BSA-soaked beads (asterisk). The right-most panel shows the magnified image of the p-AKT-positive Sertoli cells indicated by the broken square (broken line, the tubular wall). Figures in b were generated by using R^60^. *Asterisk* beads, *RT* rete testis, *SV* Sertoli valve, *ST* seminiferous tubule. Scale bars represent 50 µm.

Having identified the SV-specific AKT phosphorylation, we then asked which signaling molecule contributes to this local AKT activation. Based on our previous study showing the SV-specific HSPG (Heparan Sulfate Proteoglycan) enrichment in hamsters^26^, we hypothesized that HSPG, which functions as a reservoir and co-receptor of FGF ligands^32^, modulates the SV-specific AKT activation through its local storage and reception of ligands produced by the RT. To test this hypothesis, we focused on FGFs as a potential upstream of AKT activation at the SV. We first confirmed the enrichment of HSPG in the basement membrane of the SV region in adult wild-type mouse testis (Fig. 3a), which is consistent with our previous work in hamster. HSPG was observed predominantly in the SV region, while the RT region marked by ECAD (E-cadherin, also known as Cadherin-1) immunoreactivity only showed little expression of HSPG (Fig. 3a’). We then examined the gene expression levels of FGF ligands and their receptors in RT, SV, and convoluted ST of both wild-type ICR mice and germ cell-depleted mutant (*W/W*^*v*^) mice. To collect RT, SV, and ST samples for genetic analysis, we first injected trypan blue solution into ICR and *W/W*^*v*^ mouse testes via efferent ducts to facilitate the visualization of RT and SV regions, and then manually isolated the RT, SV, and distal convoluted ST fragments separately under a dissecting microscope (n = 4 for ICR mice, n = 5 for *W/W*^*v*^ mice). qRT-PCR analyses confirmed that the expression levels of *Fgf9* and *Fgf10* were significantly higher in the RT compared to the convoluted ST in wild-type mice (Fig. 3b). The expression levels of *Fgf9* were significantly higher in the RT also in the *W/W*^*v*^ mutant mice, suggesting that its region-specific difference is not due to the existence of germ cells (Fig. 3b). Next, we examined the mRNA expression levels of two major FGF receptor genes in Sertoli cells, *Fgfr1* and *Fgfr2*^33^. As a result, *Fgfr2* was expressed significantly higher in the SV region compared to the ST region both in *W/W*^*v*^ and wild-type mice (Fig. 3b). *Fgfr1* expression in the SV was significantly higher in the *W/W*^*v*^ mice, but not in wild-type mice potentially due to the contamination of germ cells (Fig. 3b). We further performed in situ hybridization in the wild-type mice to confirm the localization of mRNA in each gene. As a result, the expression of *Fgf9* was detected in the RT region, while hardly any *Fgf9* signals were observed in SV and ST regions (Fig. 3c). On the other hand, both *Fgfr1* and *Fgfr2* mRNA were detected both in the SV and RT regions (Fig. 3c). Such co-expression of HSPG and FGFRs in the SV region suggests the potential binding and activation of FGFs within the SV region.

To investigate the correlation between FGF signaling and AKT phosphorylation at the SV region, we next examined the effect of exogenous FGF9 on the p-AKT signals in convoluted seminiferous tubules by using an in vivo bead transplantation assay (n = 5)^13,34^. In this experiment, *W/W*^*v*^ mutant mice were used to exclude the effect of FGF9 on germ cells. Even without the germ cells, p-AKT signals were observed sporadically in the convoluted seminiferous tubules of the *W/W*^*v*^ mutant mice (Fig. S2a). However, the signal intensity of such p-AKT was relatively weak compared to that in the SV region (Fig. S2b), and not all the Sertoli cells within the cross-sectioned seminiferous tubules expressed p-AKT in the *W/W*^*v*^ mutant mice. In contrast, p-AKT signals were greatly increased in the convoluted ST adjacent to the FGF9-soaked beads, but not in the ST far away from the FGF9-soaked beads nor the ST adjacent to the BSA-soaked beads (Fig. 3d). Therefore, these findings suggest the possible contribution of FGF-FGFR signaling to the SV-specific expression of p-AKT.

### Identification of *Cyp26a1* as an SV-specifically upregulated gene

**Figure 4:**
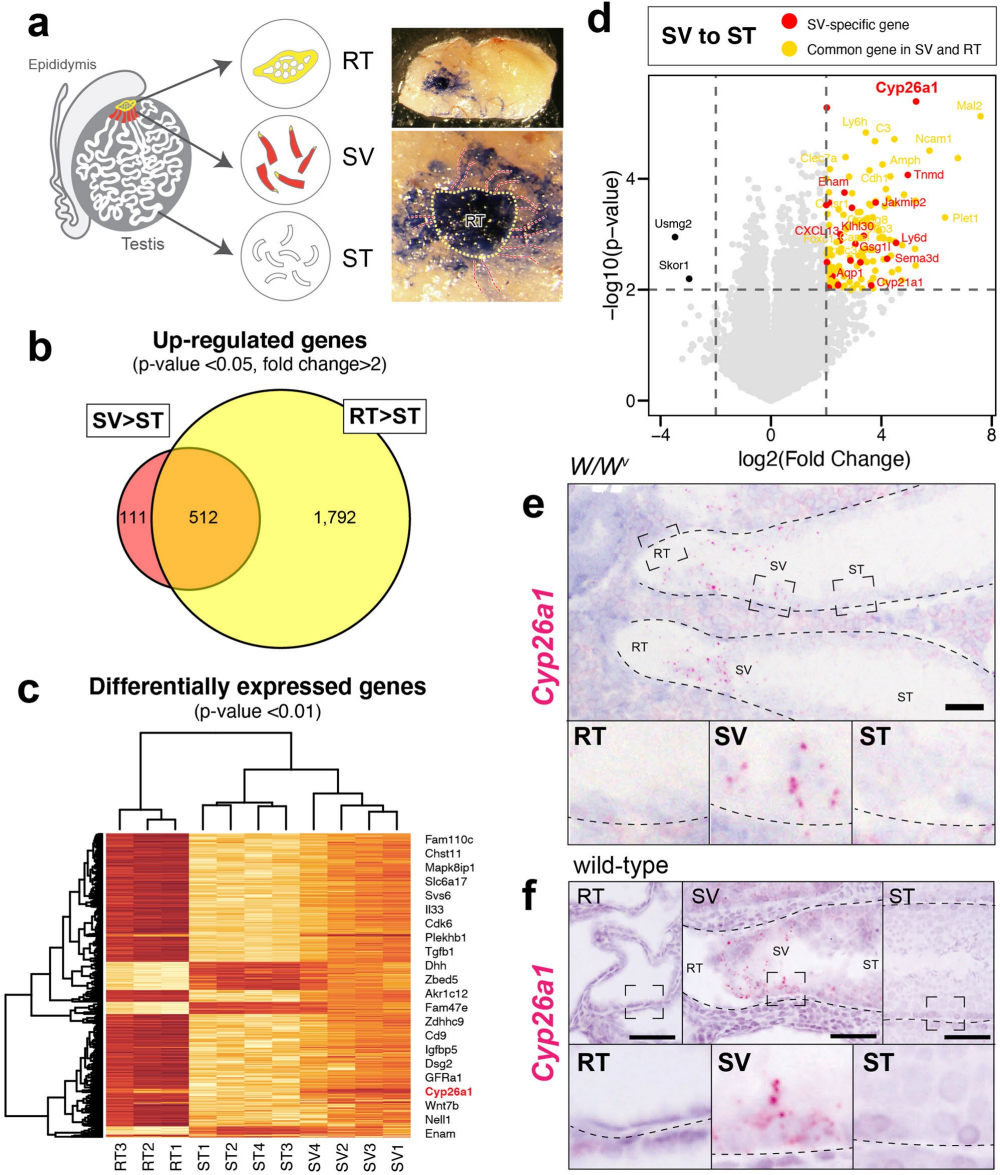
Identification of *Cyp26a1* as an SV-specifically upregulated gene. (**a**) Schematics illustration and dissecting micrographs of the RT (yellow broken line) and the SV (red broken lines) of a *W/W*^*v*^ mutant mouse testis injected with trypan blue solution through the efferent ducts. 20 testes were used for each set of microarrays, and we subjected 3 sets of RT and 4 sets of SV and ST for the microarray analysis. (**b**) Venn diagrams of the probes highly expressed in the SV (623 genes) and the RT (2,304genes) compared to the ST (> twofold changes with *p*-value < 0.05). 111 probes were exclusively expressed in the SV region, which can be regarded as the SV-specific probes. (**c**) Heatmap representation of 862 DEGs with p-value < 0.01. (**d**) Volcano plot of log_2_ Fold Change (x-axis) and -log_10_ p-value (y-axis) of the SV in comparison with the ST. Red dots represent the genes exclusively expressed in the SV, while orange dots represent the genes which also express in the RT. (**e,f**) In situ hybridization images show high *Cyp26a1* signals in the SV epithelia in the *W/W*^*v*^ (**e**) and wild-type (**f**) mouse testes. In (**e**–**f**), lower panels show magnification of the area indicated by broken rectangles in the upper panels. Figures in c and d were generated by using R^60^. Broken lines in (**e**–**f**) indicate the outlines of the ST. *RT* rete testis, *SV* Sertoli valve, *ST* seminiferous tubule. Scale bars represent 50 µm.

To further understand the regulatory mechanism of the SV region on a molecular basis, we next performed the global mRNA analyses, aiming to identify the genes specifically expressed in the SV region. The RT, SV, and convoluted ST fragments were manually separated from adult *W/W*^*v*^ mice upon intratubular trypan blue injection (Fig. 4a), and then subjected to the microarray analyses (20 testes for each set of microarrays; 3 sets for RT, 4 sets for SV and ST).

The transcriptomic analysis resulted in the identification of 623 and 2,304 upregulated probes in the SV and RT respectively, compared to the convoluted ST (> twofold change with *p*-value < 0.05). Since 512 probes were shared by the 2 groups, 111 probes were identified as SV-specific probes, which are exclusively expressed in the SV region (Fig. 4b). Gene ontology (GO) analysis of the 111 SV-specific probes suggested the terms “retinoic acid metabolic process” (*e.g.*, *Cyp26a1, Rbp1*; p = *7.4*E^–02^), “positive regulation of protein phosphorylation” (e.g., *Il34, Camp, Gpnmb, Ptpn5*; p = 6.9^–02^), “detection of mechanical stimulus involved in sensory perception” (e.g., *Asic2, Serpine2*; p = 2.5E^–02^) and “positive regulation of cell proliferation” (*e.g.*, *Il34, Sfrp1, Ctsh*; p = 2.5E^–02^). On the other hand, GO analysis of the 1792 RT-specific genes identified by microarray analyses yielded the terms “positive regulation of epithelial cell proliferation” (e.g., *Fgf1, Fgf9, Bmp4, Notch1, Igf1, Vegfa*; p = 7.8E^–06^), “Wnt signaling pathway” (e.g., *Wnt7b, Wnt9a*; p = 1.5E^–02^) and “Wound healing” (e.g., *Tgfa, Cx3cl1;* p = 4.4E^–02^). These results suggest the secretion of various signaling factors in the RT region, which may affect the terminal end of the ST to obtain its unique phenotypes. Differentially expressed gene (DEG) analysis resulted in the identification of 862 DEGs (p < 0.01, Fig. 4c). 107 DEGs were upregulated both in the SV and RT regions (orange dots in Fig. 4d), while 24 DEGs were exclusively upregulated in the SV region (red dots in Fig. 4d). Among such SV-specific DEGs, we identified *Cyp26a1* as the most significantly upregulated gene with top fold change (Fig. 4d). Since *Cyp26a1* encodes a cytochrome P450 enzyme which controls the metabolic inactivation of RA^35^, SV-specific expression of *Cyp26a1* implies unique RA metabolization within the SV region. Being consistent with the microarray data, in situ hybridization confirmed the region-specific expression of *Cyp26a1* in the Sertoli cells located within the SV region of *W/W*^*v*^ mutant mice (Fig. 4e). The expression of *Cyp26a1* was also observed in the SV region of wild-type mice (Fig. 4f). These data indicate constitutively high *Cyp26a1* expression in the Sertoli cells within the SV region irrespective of the presence of spermatogenic cells, and in turn suggest the constitutively active degradation of RA in the SV region.

### Exogenous RA treatment induces ectopic c-KIT-positive spermatogonial patches and subsequent disruption of the SV structure

**Figure 5:**
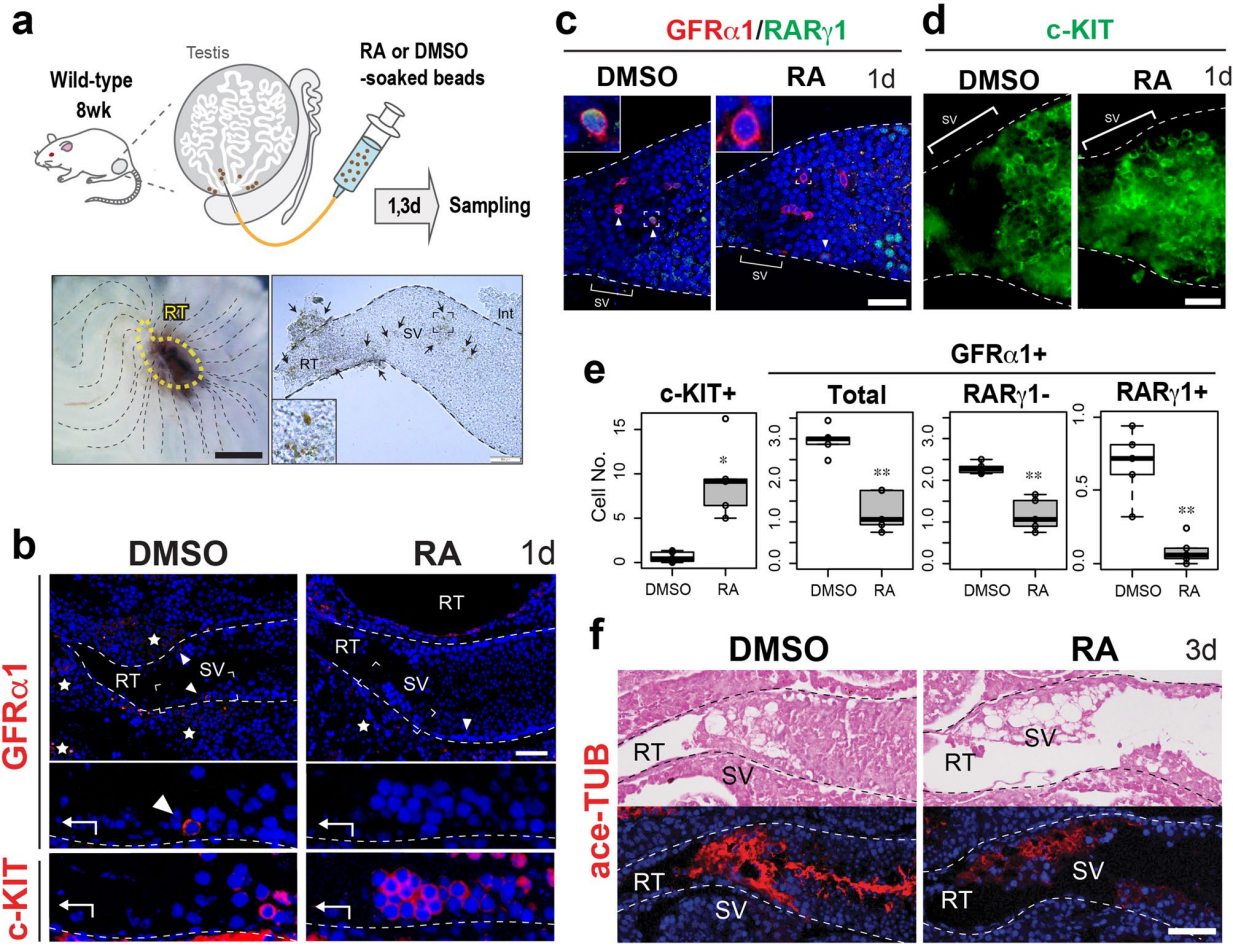
RA-induced ectopic appearance of A_diff_ patches in the SV, leading to structural alterations of the SV epithelia of wild-type mouse testes. (**a**) Schematic representation of RA or DMSO (control)-soaked beads treatment. Left bottom panel shows the gross morphology of the testis removed of tunica albuginea, transplanted with the beads (brown) locally around the SV region, beneath the RT region indicated by the yellow broken line. Right bottom panel shows the image of an isolated SV fragment, around which the beads are attached (brown, arrows). The phenotypes of the SV epithelia were examined at day 1 (**b**–**e**) and day 3 (**f**) after RA treatment (n = 4 each). (**b**) Anti-GFRα1 (red, arrowheads) and anti-c-KIT (red) staining of the serial sections of the SV region, showing an ectopic appearance of a c-KIT-positive A_diff_ within the SV epithelia of RA-treated testes. (**c**–**e**) Whole mount immunohistochemistry of anti-GFRα1 (cell surface staining, red)/RARγ1 (nuclear staining, green) (**c**) and anti-c-KIT (green; **d**) in the SV region, showing the disappearance of GFRα1/RARγ1-double positive cells (arrowheads) and the ectopic appearance of c-KIT-positive A_diff_ patches in the proximal SV region (upper left insets in **c**, higher magnified images of the GFRα1-positive cell indicated by the broken square). In (**e**), box plots show significant alterations of c-KIT and GFRα1-positive (total, RARγ1-positive and -negative) cell number within the proximal SV region (0 ~ 50 μm from RT; n = 5; *p < 0.05; **p < 0.01). Analysis was performed using unpaired Student’s t-test. (**f**) HE staining and ace-TUB (red) immunohistochemistry of the SV region at day 3 after RA treatment showing the disrupted ace-TUB signals in the SV region. Broken lines indicate the outlines of the ST. Images in c were taken by confocal microscopy. Insets in (**a**) and (**c**) and lower panels in (**b**) indicate the magnification of the region surrounded by broken rectangles in each panel. Figures in (**e**) were generated by using R^60^. Stars in (**b**), non-specific signals in the interstitium. *Int* interstitial tissue, *RT* rete testis, *SV* Sertoli valve, *ST* seminiferous tubule. Scale bars represent 500 µm in the left panel of a, and 50 µm in the right panel of (**b**–**d**) and (**f**).

Finally, we examined the effect of exogenous RA on the structure and function of the SV region in wild-type mouse testes (Fig. 5a–f). In this experiment, we transplanted RA-soaked microbeads locally around the SV region to temporarily increase the local RA levels (Fig. 5a). Being consistent with our previous report^26^, control (DMSO-treated) mouse testes had hardly any c-KIT-positive A_diff_ within the proximal part of the SV region (0 ~ 50 μm from the edge of rete testis), where GFRα1-positive/c-KIT-negative A_undiff_ were constitutively maintained (Fig. 5b). On the other hand, RA-treated mouse testes showed a reduced number of GFRα1-positive A_undiff_ within the proximal SV region (Fig. 5b,c; “GFRα1 + Total” in Fig. 5e), while c-KIT-positive A_diff_ were enriched within the SV region at day 1 after the RA treatment (Fig. 5b,d; “c-KIT + ” in Fig. 5e). Since RARγ-positive subpopulation was previously shown to maintain the differentiation competence of GFRα1-positive spermatogonia^15,36^, we then quantified the RARγ-negative/positive subpopulation of GFRα1-positive cells in the proximal SV region treated with RA and DMSO respectively. As a result, we found a drastic reduction of RARγ-positive subpopulation (13.0% of the control value) together with a mild reduction of a RARγ-negative subpopulation (51.4% of the control value) in the RA-treated group (n = 5, two right graphs in Fig. 5e). In contrast, RA treatment did not seem to affect the expression of GDNF and CyclinD1 (CCND1) in the Sertoli cells located within the SV region (Fig. S3), which are other characteristics of the SV region^26^. These findings suggest that the c-KIT-positive spermatogonial patches observed at the proximal SV region in the RA-treated mice may have resulted directly from the A_undiff_-A_diff_ transition of pre-existing GFRα1/RARγ-double positive spermatogonia by the exogenous exposure to excess RA. Interestingly, in some severely affected SVs at day 3 after RA treatment, the ace-TUB-positive valve-like structures were disrupted (Fig. 5f). These data suggest that low RA levels in the SV region support the maintenance of the local A_undiff_, as well as contributing to the physical valve-like structure.

## Discussion

In this study, we demonstrated the non-cell-autonomous regeneration of the SV epithelia by taking advantage of the AMH-Treck Tg system. Although the transplanted Sertoli cells were derived from the convoluted ST, donor-derived Sertoli cells colonized in the presumptive SV region (i.e., within 200–300 µm from the RT) regenerated not only the valve-like structure, but also expressed several SV markers such as p-AKT and ace-TUB, similar to the SV epithelia in the normal intact testes (Fig. 2f). Consistent with the previous observations in wild-type mouse testis^26^, spermatogenesis was repressed in such regenerated SV epithelia (Fig. 2c–f), while patches of active spermatogenesis were frequently observed in the convoluted STs (“ST” in Fig. 2c–f). Although the function of the regenerated SV region as a stem/progenitor cell niche remains unclear, our data suggest that the SV region is non-cell autonomously specified by the Sertoli cells adjacent to the RT. In conjunction with the data showing the upregulation of p-AKT and ace-TUB in the Sertoli cells adjacent to the RT in P7 testes, prior to the formation of the valve-like structure (Fig. 1f), we speculate that the regionalization of the SV epithelia is induced and/or maintained, together with the activation of the AKT signaling pathway and tubulin acetylation, presumably by local soluble factors derived from the RT and its surrounding tissues at the terminal segment of the ST. In this study, we revealed the constitutively high expression of p-AKT in the SV epithelia, together with seminiferous epithelial cycle stage-dependent expression of p-AKT in the convoluted ST. This is reminiscent of the expression pattern of phosphorylated signal transducer and activator of transcription-3 (STAT-3), which shows constitutively high expression in the SV epithelia with some local expression in the convoluted ST at seminiferous epithelial cycle stages I–VI^29^. The onset of p-STAT3 activation in the SV Sertoli cells takes place in P7 testis^29^, coinciding with the onset of p-AKT expression (Fig. 1f), suggesting that the regionalization of the SV region takes place around P7 in the postnatal testicular development.

In the fetal mouse testes, FGF9 was previously shown to induce the proliferation of Sertoli cells through FGFR2^37–39^. FGF9, as well as FGF10, is upregulated in a testis-specific manner by 11.5 dpc and contributes to synchronous testiculogenesis of the pre-Sertoli cells in the fetal testes^40^. Concomitantly with the onset of *Fgf9* expression, testis-specific AKT activation also takes place in presumptive pre-Sertoli cells at 11.5 dpc^41^, suggesting a potential association between FGF9 signals and p-AKT activation in the developing fetal testes. The present study demonstrated the increased p-AKT signals in the Sertoli cells of convoluted ST caused by exogenous FGF9 (Fig. 3d). Since FGF9 is known to activate the PI3K/AKT pathway as well as MEK/ERK pathway^42^, RT-derived FGF9 can be associated with the activation of AKT signals in the Sertoli cells within the SV region. Interestingly, the basement membrane at the SV region has the region-specific enrichment of HSPGs, which works as a reservoir for several FGF ligands^31^. Therefore, it might be possible that the RT-derived FGFs bind to the HSPGs at the SV region and subsequently cause signal transduction via local FGFRs. Since FGF9 is known to work over a short-range^32^, and the SV region is adjacent to the RT, we assume that Sertoli cells in the SV can perceive the RT-derived FGF9. Taken together, RT-derived FGF ligands and their local perception through the HSPG can be one of the causes for the activation of AKT within the SV region. Microarray analyses revealed the high expressions of various cytokine and growth factor genes in RT, including those encoding candidate ligands upstream of AKT/STAT3 signaling, such as FGF9, suggesting their potential contribution to non-cell autonomous specification and regionalization of the SV epithelia in vivo. Meanwhile, our microarray analysis indicated the expression of various cytokines and growth factor genes in the RT region aside from FGF9, which can also be candidate ligands upstream of AKT/STAT3 signaling. Therefore, a further study on such ligands is required to comprehensively understand the RT-derived stimuli, which play a role in the non-cell autonomous regionalization and the constitutive p-AKT/p-STAT3 activation of the SV epithelia in vivo.

It is traditionally known that FGFs and RA signaling acts in an opposing manner in the regionalization of various organs such as limb, neural pattern, segmentation, and somite formation^43–45^. In fetal and postnatal testes, FGF9 antagonizes RA-dependent meiotic differentiation of germ cells in part by maintaining *Nanos2* expression^7,8,46^. In adult testes, FGFs including FGF2 and FGF5 maintain the self-renewal of stem/progenitor spermatogonia independent of GDNF signals^14,47^, while RA deficiency supports the A_undiff_ to retain their stem/progenitor state in vivo^1,4,17^. FGF9, as well as FGF2, maintains these stem/progenitor spermatogonia in vitro^45^. FGFs including FGF5 were shown to be produced by the lymphatic endothelial cells around the seminiferous tubule to support A_undiff_^14^. Considering that lymphatic vessels from convoluted STs join together around RT and SV regions^48^, the source of the FGF ligands in the RT can be its epithelial cells as well as its surrounding lymphatic tissue. Similarly, excess expression of a niche factor, GDNF, represses the differentiation of A_undiff_, leading to inactive spermatogenesis in vivo^11,34,49,50^. Since GDNF is also highly expressed in the SV region^26^, GDNF and FGFs may contribute to the structure of SV epithelia by suppressing the differentiation of local A_undiff_ in rodent testes.

In mammalian spermatogenesis, RA is involved in (1) the A_undiff_–A_diff_ transition of spermatogonia, (2) spermiation, and (3) regulation of the seminiferous epithelial cycle^18^. RA has been reported to be dispensable for the meiotic initiation of the germ cells^51–53^, whereas injection of exogenous RA promotes the differentiation of A_undiff_ in vivo^54^. Similarly, in our study, local administration of exogenous RA promoted the ectopic appearance of c-KIT-positive A_undiff_ in the SV region. Together with the SV-specific expression of Cyp26a1, it is reasonable to speculate that the levels of RA in the SV region are maintained low, preventing the A_undiff_–A_diff_ transition of the local spermatogonia within the SV region. Considering that another RA metabolizing enzyme, *Cyp26b1*, expresses in the peritubular myoid cells to block the entry of interstitial RA into the seminiferous tubules^55,56^, *Cyp26a1* in the SV region may also function as a local catabolic barrier insulating the A_undiff_ from RT-derived RA. Since *Cyp26a1*-null mutant mouse testes were reported to have no appreciable defects in the convoluted ST^35^, the compensational roles of *Cyp26a1* and *Cyp26b1* in the SV region cannot be neglected, yet their detailed phenotypes of the SV remain unclear. Meiotic germ cells express *Aldh1a1-3,* which make the germ cell one of the major sources of RA in the testis^54^, and the lack of meiotic germ cells in the SV region can also be a cause for the low level RA within the SV region. Although RA is synthesized by both Sertoli cells and germ cells, germ cell-derived RA has been reported to play important roles in the A_undiff_–A_diff_ transition of spermatogonia at the initial onset of spermatogenesis^54^. Taken together, the lack of meiotic germ cells within the SV region contributes to its local low RA signaling state, in combination with the local degradation of RA by *Cyp26a1* expression.

**Figure 6:**
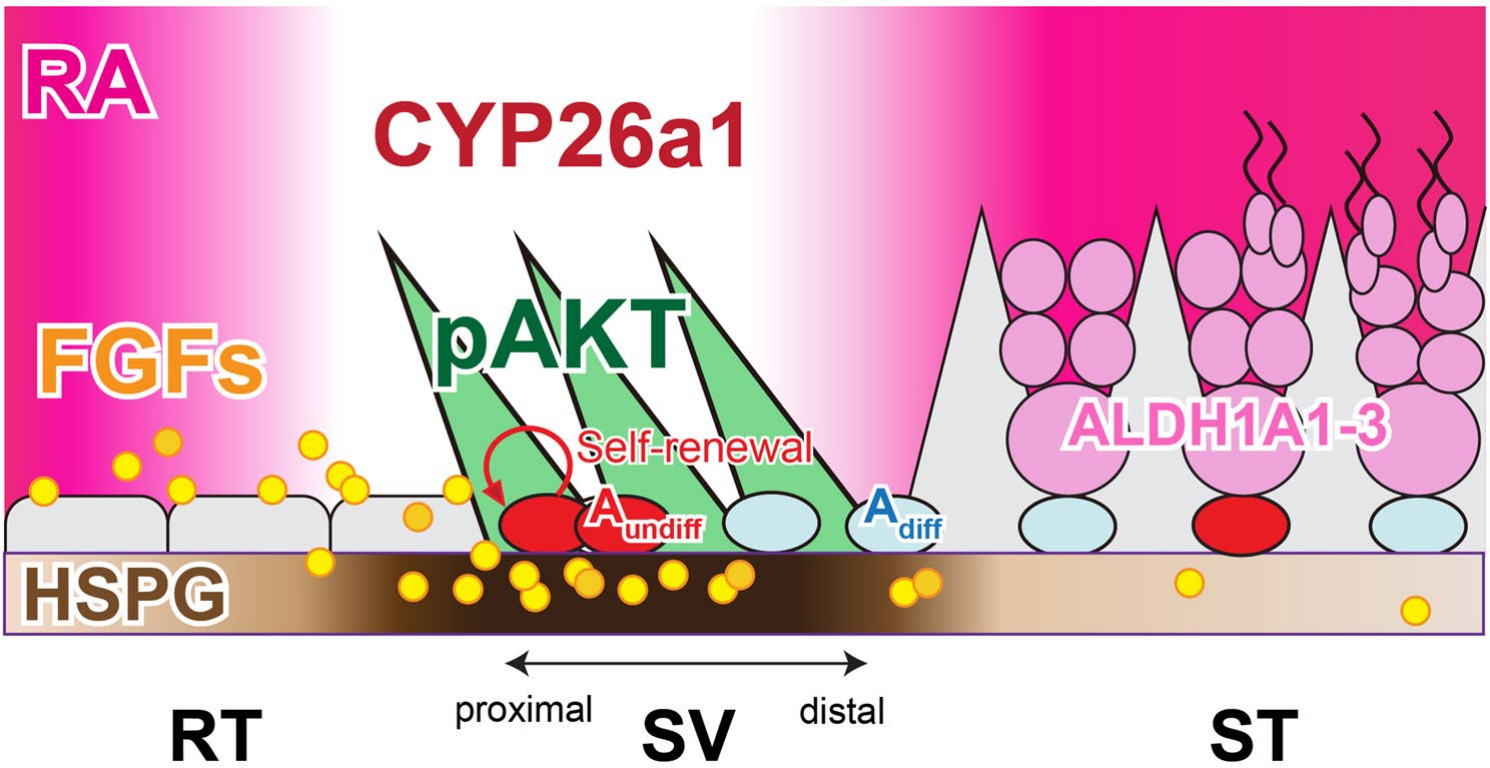
Low RA levels and RT-derived factors may mediate the non-cell autonomous regionalization of the SV epithelia. A model schematic illustrating a potential contribution of RT-derived FGFs and local low-level RA to the regionalization of the SV epithelia. Through the region-specifically enriched HSPG in the SV region, FGFs derived from the RT (e.g., FGF9) get immobilized and perceived in the SV region, and then possibly mediate the constitutive AKT phosphorylation in the SV Sertoli cells. Such RT-derived FGF ligands can also contribute to the maintenance of stem/progenitor cell population in the SV region by promoting their self-renewal. On the other hand, SV-specific expression of CYP26A1 degrades RA within the SV region to maintain the local RA at low levels, repressing the A_undiff_–A_diff_ transition of stem/progenitor cells. The absence of ALDH1A1A1-3 expressing meiotic germ cells within the SV region may also contribute to the physical maintenance of the valve-like structures, as well as the low-RA levels within the SV region.

In our proposed model, the specification and regionalization of the SV epithelia are non-cell autonomously mediated by the factors derived from the RT, resulting in the AKT phosphorylation in the SV Sertoli cells (Fig. 6). Particularly, FGFs (e.g., FGF9) may contribute to the maintenance of A_undiff_ in the SV region, through its region-specific enrichment of HSPG in the basal lamina. Moreover, the high expression of *Cyp26a1* in the SV region leads to the region-specific low-RA signaling states, which may promote the maintenance of GFRα1-positive A_undiff_ and repress their differentiation into c-KIT-positive A_diff_. A lack of differentiated germ cells (i.e., c-KIT-positive A_diff_ and meiotic/post-meitoic germ cells) may also physically allow the formation of unique Sertoli cell morphology within the SV region, extending the cytoplasmic processes into the adluminal compartment to form the valve-like structure within the SV region. In this study, exogenous RA treatment induced the ectopic appearance of c-KIT-positive A_diff_ patches within the SV, which could be differentiated from the pre-existing GFRα1/GFRγ1-double positive A_undiff_ within the SV region or recruited from the proximal ST region adjacent to the SV. Such aberrant accumulation of c-KIT-positive cell patches protruding into the luminal side might have contributed to the disruption of ace-TUB-positive SV structure. Considering the potential contribution of meiotic germ cell-derived RA for the initial A_undiff_-A_diff_ progression of the stem/progenitor cell population^50^, the lack of advanced spermatogenic cells within the SV region may be also crucial for the physical and endocrinological maintenance of the SV epithelia.

Although the existence of the SV region has been recognized for half a century, the majority of its functionality and molecular mechanisms remain unknown. This study provided the first direct evidence of non-cell autonomous regionalization of mouse SV epithelia, as well as its unique gene expression profile. A further study on SV-specific genes may resolve the molecular mechanisms underlying the functional and biological significances of the SV region, as well as the mammalian stem/progenitor cell niche.

## Methods

### Animal care and use

C57BL/6-Tg *AMH-Treck* mice (AMH-Treck mice)^31^, C57BL/6-R26-H2B-mCherry knock-in mice (H2B-mCherry mice)^57^ and C57BL/6-Tg (CAG-EGFP) mice (GFP mice; SLC Japan) were used for Sertoli cell transplantation experiments. Wild-type mice (C57BL/6 and ICR mice) and *W/W*^*v*^ mice (SLC Japan) were used for in situ hybridization, immunohistochemistry, microarray, and quantitative reverse transcription-polymerase chain reaction (qRT-PCR) analyses. All animal experiments were performed in accordance with Guidelines for Animal Use and Experimentation at the University of Tokyo, and all the experimental procedures herein performed were approved by the Institutional Animal Care and Use Committee, Graduate School of Agricultural and Life Sciences, The University of Tokyo (approval IDs, P13-762 and P13-764).

### Sertoli cell transplantation

For Sertoli cell transplantation, AMH-Treck (3 ~ 4 weeks old) and GFP/mCherry (1 week old) male mice were used as recipients and donors, respectively^31^. First, AMH-Treck male mice were treated with diphtheria toxin (DT; Sigma-Aldrich, 4 μg/kg i.p.) 4 days before transplantation. To obtain donor Sertoli cells, the convoluted STs were isolated from the distal two-thirds of a whole testis (*i.e.*, the testes without RT, SV, and proximal ST) of GFP/mCherry mice. A single-cell suspension (1.0 × 10^7^ cells/mL) including immature Sertoli cells was prepared by two-step enzymatic digestion as previously reported^31^ and transplanted through the efferent ducts into the testes of DT-pretreated AMH-Treck recipients (Fig. 2a). On days 10 and 45 after transplantation, all recipient testes were analyzed for histology and immunohistochemistry. The proportion of donor-derived cells (relative number of mCherry-positive cells per total cells) were 96.9 ± 0.5% in Sertoli cells and 75.2 ± 15.5% in germ cells on 45 days after transplantation (n = 4).

### In vivo treatment of exogenous FGF9 and retinoic acid

For FGF9 treatment, FGF9 (R&D systems, 0.1 mg/ml)-soaked beads (Affi-gel blue, Bio-Rad, ~ 50  μm) were injected into the interstitial regions of *W/W*^*v*^ mouse testes as previously reported^13,34^. The beads were labeled with DiI prior to the transplantation, which makes it distinguishable in the section. At 2 ~ 24 h after the bead treatment, testes were isolated for the subsequent histology and immunohistochemical analyses.

For RA treatment, wild-type male mice (ICR, 7–8 weeks old) were injected with beads (BioMag Amine) soaked in 40.0 mg/ml all-trans RA (Sigma-Aldrich) in 16% (vol/vol) dimethyl sulfoxide (DMSO) or DMSO (Fig. 5a). In this experiment, the beads were injected locally around the SV region (Fig. 5a) to examine the effects of locally elevated RA levels on the SV function. At 1 and 3 days after the treatment, testes were isolated for subsequent histology and immunohistochemical analyses.

### Immunohistochemistry and morphometry

The testes were fixed in 4% paraformaldehyde (PFA) and routinely embedded in paraffin or OCT compound. Cryosections (8 µm thickness) or deparaffinized sections (5 µm thickness) were subjected to immunohistochemical staining as described previously^58^. In brief, the sections were incubated with anti-acetylated tubulin (ace-TUB; T6793; 1:200 dilution; Sigma-Aldrich), anti-AKT (pan) (#4691; 1:300 dilution; Cell Signaling Technology), anti-CCND1 (cyclin D1; 1:100 dilution; Thermo scientific); anti-GATA4 (sc-1237; 1:75 dilution; Santa Cruz Biotechnology), anti-GDNF (sc-328; 1:100 dilution; Santa Cruz Biotechnology), anti-GFP (598; 1:200 dilution; MBL), anti-GFRα1 (AF560; 1:100 dilution; R&D Systems), anti-HSPG (10E4; 370,255–1; 1:100 dilution; Amsbio), anti-mCherry (ab125096; 1:100 dilution; Abcam), anti-kit oncogene (c-KIT; AF1356; 1:100 dilution; R&D Systems), anti-MVH/DDX4 (ab13840; 1:1,000 dilution; ab13840; Abcam), anti-phosphorylated AKT (Ser473) (p-AKT; #4060; 1:50 dilution; Cell Signaling Technology), anti-phosphorylated-p44/42 MAPK (Thr202/Tyr204) (p-Erk; #4370; 1:200 dilution; Cell Signaling Technology), anti-phosphorylated-p38 (Thr180/Tyr182) (p-p38; #4511; 1:500 dilution; Cell Signaling Technology), anti-phosphorylated-SAPK/JNK (Thr183/Tyr185) (p-JNK; #4668; 1:50 dilution; Cell Signaling Technology) and anti-SOX9 (#AB5535; 1:1000 dilution; Millipore) antibodies. Reactions were visualized by incubation with Alexa-488/594 conjugated secondary antibodies (Molecular Probes) or biotin-conjugated secondary antibodies with the Elite ABC Kit (Vector Laboratories, CA). For anti-p-AKT, p-ERK, p-p38 and p-JNK primary antibodies, Alexa Fluor 488 Tyramide Signal Amplification (TSA) kit (Invitrogen) was used to visualize the reaction. In some stained sections, the seminiferous cycle stages were identified by acrosome staining using rhodamine-labeled soybean agglutinin (SBA; Vector Stain). To verify antibody specificity for phosphorylated AKT, two serial sections were pretreated with or without alkaline phosphatase from calf intestine (ALP; #47785055; Oriental Yeast) at 34 °C for 1 h before incubation with the primary antibody.

For whole-mount immunohistochemistry, SV and ST fragments were carefully isolated and fixed in 4% PFA. After methanol permeabilization, the fragments were incubated for 12 h at 4 °C with anti-c-KIT (1:100 dilution; R&D Systems), anti-GFRα1 (1:100 dilution; R&D Systems) or anti-RARγ (1:100 dilution; Cell Signaling) antibody. After washing with PBS, the samples were incubated for 2 h at room temperature with Alexa-488/594 conjugated secondary antibodies, including DAPI. Stained whole-mount samples were photographed, and the numbers of GFRα1-positive/RARγ-negative, GFRα1-positive/RARγ-positive and c-KIT-positive cell were quantified respectively in the proximal part of SVs (i.e., 0 ~ 50 μm apart from the edge of the rete testis) as previously shown^26^. In addition, in intact wild-type mice, a proportion of RARγ-positive cells out of total GFRα1-positive spermatogonia in the SV region was at 20.1 ± 7.2%, similar to 21.3 ± 7.8% in the convoluted ST region. All of the stained samples were analyzed using an Olympus fluorescence microscope (BX51N-34-FL-2) and a Leica TCS SP8 (Leica Microsystems GmbH) confocal laser microscope. In addition, any non-specific signals inside the STs were detected in sections incubated with normal IgG instead of the primary antibody (data not shown).

### Isolation of the tubular fragments from the RT, SV and ST

To visualize the RT, SV and proximal ST, trypan blue solution (0.4%; Sigma-Aldrich) was micro-injected through the efferent ducts into the testes of *W/W*^*v*^ mutant mice or wild-type ICR mice (7 weeks old) (Fig. 4a). The tunica albuginea was then removed from the testes, and the RT, SV and distal convoluted ST fragments were isolated manually by using sharp forceps and 31G needles under a dissecting microscope. RT samples were collected from the main part just beneath the tunica albuginea, and SV samples were collected as tiny, tubular fragments (< 1 mm) adjacent to the RT region. ST samples were collected from the distal part of the testis far apart from the RT and SV regions. The border of the RT and SV regions were distinguishable by their distinct tubular diameters with different intensities of the trypan blue color. Of note, as a limitation of this study, it is highly likely that SV fragments contained a certain amount of the RT-derived cells due to the continuous tubular structure of the RT and SV regions. The samples were then subjected to the RNA analyses.

### RNA extraction, microarray, and qRT-PCR analyses

Total RNA was purified from the RT, SV, and convoluted ST fragments isolated from the *W/W*^*v*^ mutant mouse testes (20 testes for each set of microarrays: 3 sets for RT, 4 sets for SV and ST), by using the RNeasy Micro Kit (Qiagen). After cDNA synthesis, biotinylated cRNA probes were synthesized using the GeneChip 3′ IVT Plus Reagent Kit (Affymetrix) and hybridized to a Mouse Genome 430 2.0 Array (Affymetrix). Data were normalized by the MAS5.0 method, and further analyzed using R software 3.2.4. Fold change values for genes were calculated as the ratio of the signal values of RT or SV regions compared with ST (control) region. Gene expression changes with > twofold alterations with *p*-value < 0.05 (Student’s t-test) were considered significant. Gene functions were annotated based on Gene Ontology (GO) terms using DAVID Bioinformatics Resources 6.7 (http://david.abcc.ncifcrf.gov/). The microarray data have been deposited in the Gene Expression Omnibus database of the National Center for Biotechnology Information (NCBI) (accession number, GSE 111884). For qRT-PCR analysis, total RNA was purified from RT, SV and ST from wild-type mice (n = 4 animals each) or *W/W*^*v*^ mice (n = 5 animals each) as described above, and then reverse-transcribed with random hexamers by using the Superscript-III cDNA synthesis kit (Invitrogen). Specific primers and fluorogenic probes for *Fgf9* (Mm00442795_m1), *Fgf10* (Mm00433275_m1), *Fgfr1* (Mm00438930_m1), *Fgfr2* (Mm01269930_m1) and *Actb* (Endogenous Control; 4352341E) were purchased from Applied Biosystems. PCR was performed using the Applied Biosystems Step One Real-Time PCR System. The relative levels of the transcripts were normalized to that of *Actb* as an endogenous reference.

### In situ hybridization

In situ hybridization was conducted as described previously^59^. The transcript levels in deparaffinized sections of *W/W*^*v*^ and wild-type testes were determined using the RNAscope Target Probe-Mm-*Cyp26a1* (468,911, NM_007811.2), *Fgf9* (499,811, NM_013518.4), *Fgfr1* (443,491, NM_010206.3), *Fgfr2* (443,501, NM_010207.2) and negative control probe *DapB* (310,043, EF191515) with the RNAscope 2.5 HD Reagent Kit-RED system (ACDBio) according to the manufacturer’s instruction.

### Statistical analyses

The qRT-PCR data were analyzed by Paired t-tests using R software^60^. Morphometric data were analyzed by Student’s t-test. A *p*-value < 0.05 was considered indicative of statistical significance. Quantitative data are presented as mean ± standard error of the mean (SEM). Throughout this study, “n” refers to the number of animals, except for the microarray analysis, in which 20 testes (10 animals) were used for each set of microarray analysis.

## Supplementary Information

Supplementary Information

## Data Availability

The authors confirm that all data underlying the findings are fully available without restriction. All relevant data are within the paper and its Supporting Information files.
