# Supplementary material for: Low retinoic acid levels mediate regionalization of the Sertoli valve in the terminal segment of mouse seminiferous tubules
Source: Sci Rep. 2021 Jan 13;11:1110. doi: 10.1038/s41598-020-79987-4 (PMC7806815; doi:10.1038/s41598-020-79987-4)
Supplement: Supplementary file 1 — Supplementary Information [file 41598_2020_79987_MOESM1_ESM.pdf]

## Supplementary Information

# Low Retinoic Acid Levels Mediate Regionalization of The Sertoli Valve in The Terminal Segment of Mouse Seminiferous Tubules

Kasane Imura-Kishi[1, #], Aya Uchida[1, #], Naoki Tsunekawa[1], Hitomi Suzuki[2], Hinako M. Takase[2], Yoshikazu Hirate[2], Masami Kanai-Azuma[2], Ryuji Hiramatsu[1], Masamichi Kurohmaru[1], Yoshiakira Kanai[1, *]

[1]Department of Veterinary Anatomy, The University of Tokyo, Bunkyo-ku, Tokyo, Japan;

[2]Department of Experimental Animal Model for Human Disease, Center for Experimental Animals, Tokyo Medical and Dental University, Bunkyo-ku, Tokyo, Japan

[#]The first two authors contributed equally to this work.

*Correspondence: aykanai@mail.ecc.u-tokyo.ac.jp

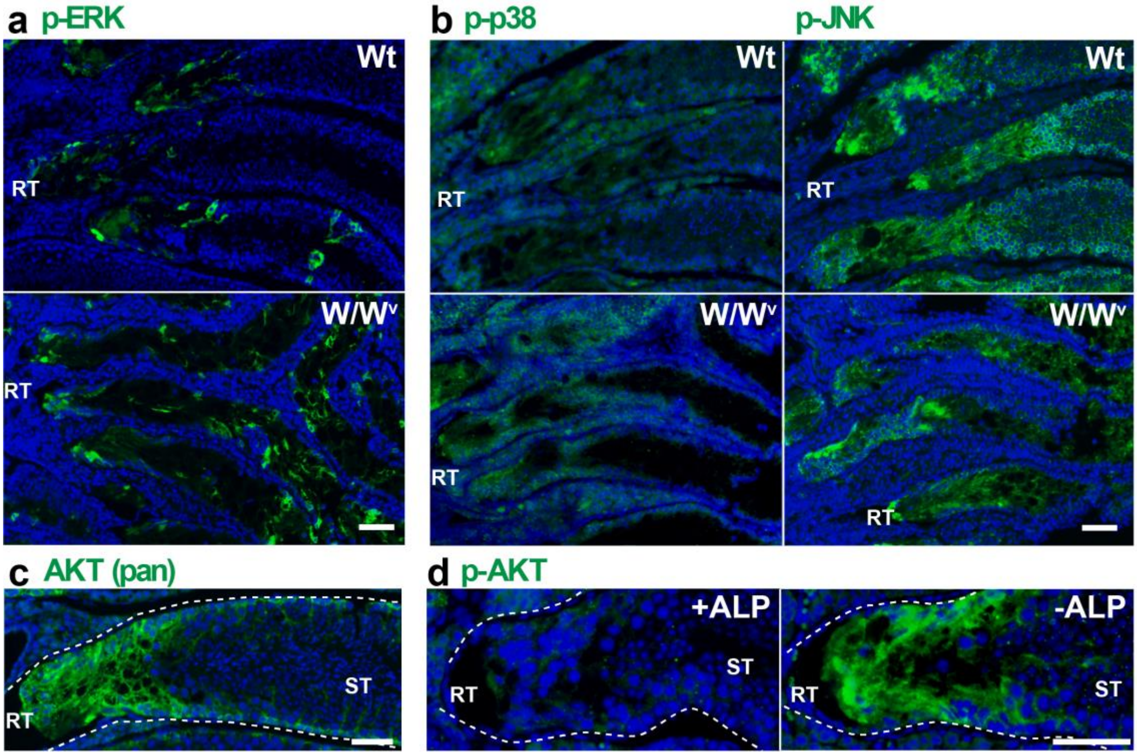


**Fig. S1. Immunostaining of phosphorylated ERK, p38, and JNK in the proximal region of wild-type and *W/W^v* testes**

**(a, b)** Anti-p-ERK, anti-phosphorylated p38 (p-p38), and anti-phosphorylated JNK (p-JNK) staining (green) show no region-restricted expression in the SV. Expression levels of p-p38 were relatively low in wild-type (Wt) and *W/W^v* testes. **(c)** Anti-AKT (pan) staining (green) of wild-type testes, showing ubiquitous AKT expression in the SV and convoluted seminiferous epithelia. **(d)** Anti-p-AKT immunostaining of two serial sections of wild-type testis pretreated with or without ALP. p-AKT signals in the SV region (green) are reduced by pretreatment with ALP. Broken lines are the outlines of the ST. ALP, alkaline phosphatase; RT, rete testis; ST, seminiferous tubule. Scale bars represent 50 μm.

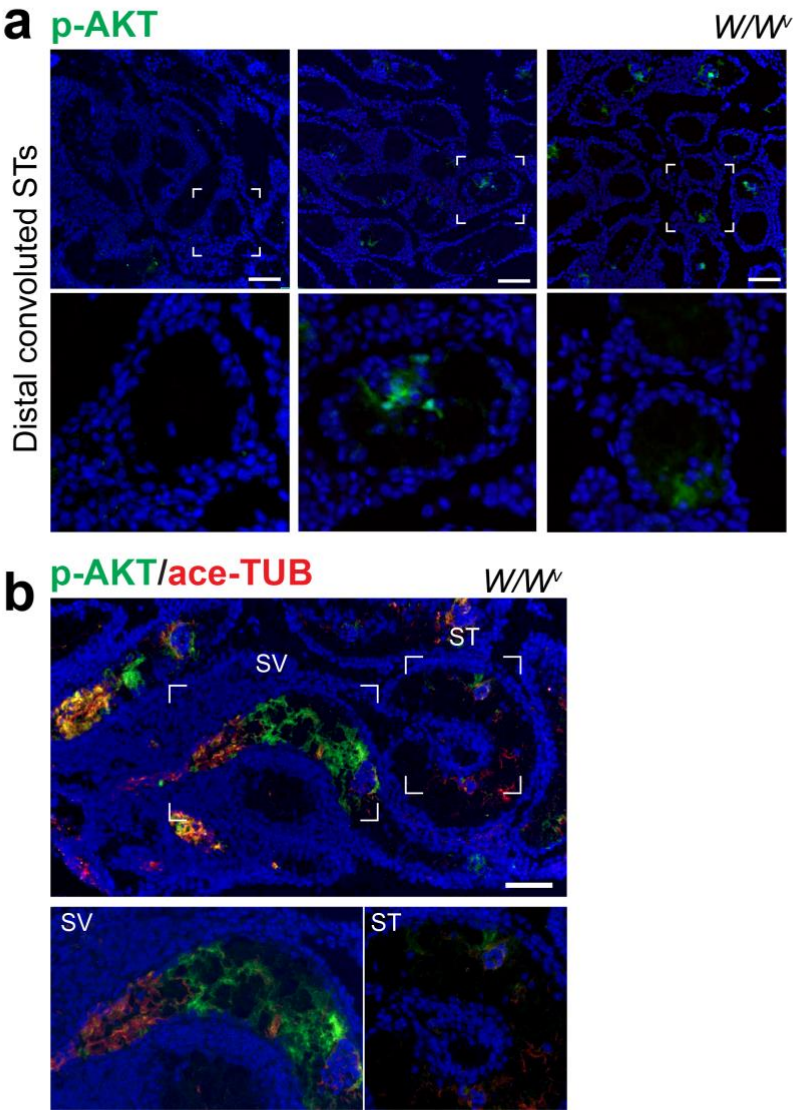


**Fig. S2. Immunostaining of p-AKT in the convoluted STs of adult *W/W^v* mouse testis (a, b)** Anti-p-AKT staining (green in a, b) and anti-ace-TUB staining (red in b), showing sporadic expression patterns of p-AKT in the Sertoli cells of convoluted STs (a, b), in contrast to high-level constitutive expression of p-AKT within/near the ace-TUB-positive SV regions (b). In a, three panels represent the convoluted STs from distal parts of the testis of three independent *W/W^v* mice. Lower panels in a and b show magnification of the region surrounded by the broken rectangle in the upper panels. SV, Sertoli valve; ST, seminiferous tubule. Scale bars represent 200 μm.

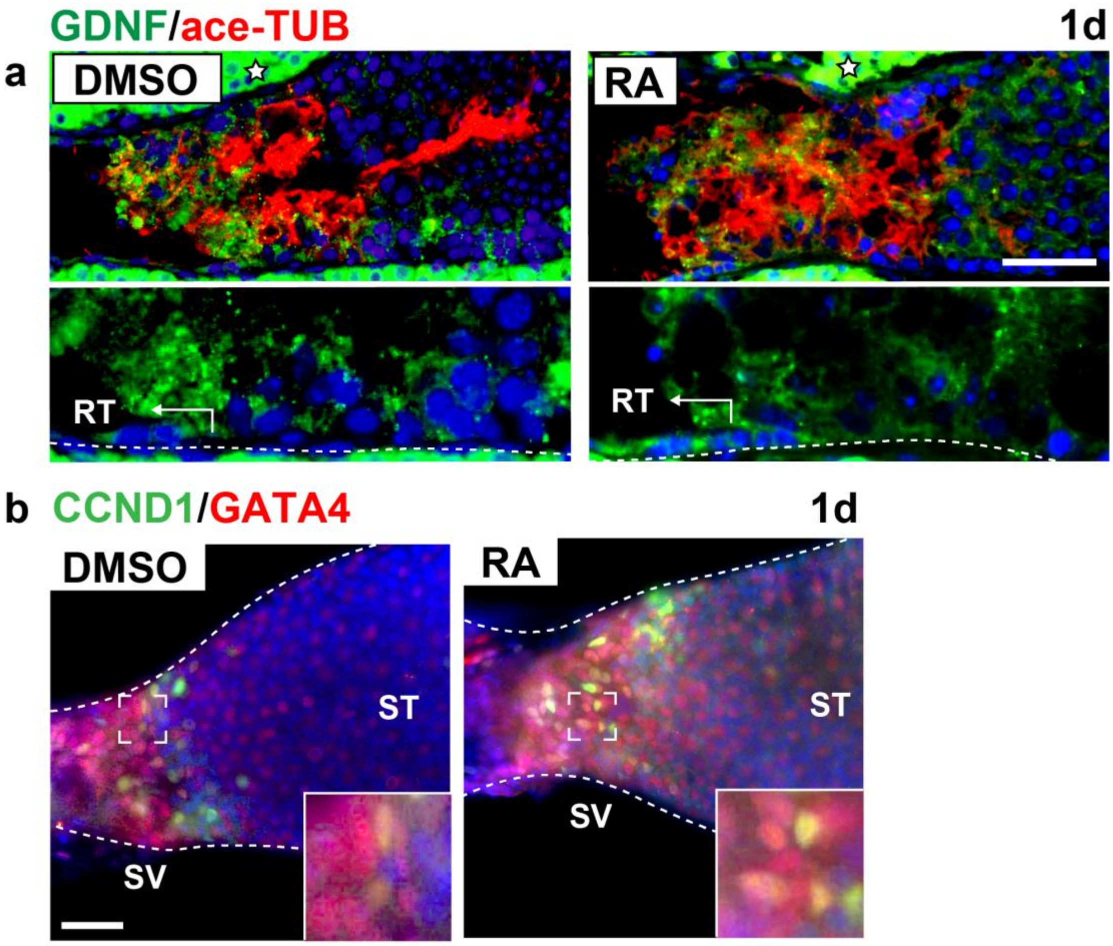


**Fig. S3. No appreciable influences of exogenous RA treatment in anti-GDNF and anti-CCND1 signal intensities in the SV epithelia.**

(**a**) Anti-GDNF (green)/ace-TUB (red) immunostaining of the sagittal sections of the SV region (at day 1 after the DMSO and RA treatment), showing no appreciable difference in GDNF expression in the ace-TUB-positive SV region between the DMSO- and RA-treated samples. Lower panels of green channel show the higher magnified images of the SV epithelia shown in the upper panels. (**b**) Anti-CCND1 (green)/anti-GATA4 (red) staining of the whole-mount SV samples at day 1 after the DMSO or RA treatment, showing no appreciable influences on the CCND1-positive signals in the GATA4-positive Sertoli cells within the SV region. Insets in b show magnification of the region surrounded by the broken rectangle in each panel. Broken lines are the outlines of the ST. Star, non-specific signals in the interstitium; SV, Sertoli valve; ST, seminiferous tubule. Scale bars represent 50 μm.
